# Multisystem FDG-avid sarcoidosis with osseous involvement mimicking disseminated metastatic malignancy

**DOI:** 10.1093/omcr/omag165

**Published:** 2026-09-02

**Authors:** Jamil Wafi, Mohammed Alkhanafsa, Soud Deek, Aya Alqattaa, Mohammed Alkhatib, Bara Wazwaz, Irfan Ul Haq

**Affiliations:** Internal Medicine Department, Hamad General Hospital, Hamad Medical Corporation, Al Rayyan Road, Doha, P.O. Box 3050, Qatar; Internal Medicine Department, Hamad General Hospital, Hamad Medical Corporation, Al Rayyan Road, Doha, P.O. Box 3050, Qatar; Internal Medicine Department, Hamad General Hospital, Hamad Medical Corporation, Al Rayyan Road, Doha, P.O. Box 3050, Qatar; Internal Medicine Department, Islamic University of Gaza, Al-Thalathini Street, Al-Rimal, Gaza City, P.O. Box 108, Palestine; Pulmonary Department, Hamad General Hospital, Hamad Medical Corporation, Al Rayyan Road, Zone 37, Doha, P.O. Box 3050, Qatar; Pathology Department, Hamad General Hospital, Hamad Medical Corporation, Al Rayyan Road, Zone 37, Doha, P.O. Box 3050, Qatar; Pulmonary Department, Hamad General Hospital, Hamad Medical Corporation, Al Rayyan Road, Zone 37, Doha, P.O. Box 3050, Qatar

**Keywords:** sarcoidosis, FDG-PET, pulmonary embolism, osseous sarcoidosis, multisystem disease

## Abstract

Sarcoidosis may closely mimic disseminated malignancy on FDG-PET/CT because active granulomatous inflammation can demonstrate intense multisystem FDG uptake. We report a 59-year-old woman with previously diagnosed pulmonary sarcoidosis who presented with acute dyspnea and right leg pain and was found to have segmental and subsegmental pulmonary emboli on CT pulmonary angiography. FDG-PET/CT demonstrated extensive intensely hypermetabolic mediastinal, hilar, abdominal and pelvic lymphadenopathy with pulmonary, hepatic, splenic and multifocal osseous involvement, including a right iliac lesion (SUVmax 12.3), raising strong suspicion for disseminated metastatic malignancy or lymphoproliferative disease. CT-guided biopsy of the iliac lesion revealed non-necrotizing granulomatous inflammation without evidence of malignancy or infection, confirming multisystem sarcoidosis with osseous involvement. This case highlights the importance of histological confirmation when FDG-PET/CT findings in sarcoidosis mimic metastatic disease.

## Introduction

Sarcoidosis is a multisystem granulomatous disease that may demonstrate intense FDG uptake on PET/CT, occasionally mimicking disseminated malignancy or lymphoma [1, 2]. Osseous involvement is uncommon and may further complicate radiological interpretation because imaging findings often resemble skeletal metastases [3].

## Case presentation

A 59-year-old woman, lifelong non-smoker with a history of hypertension and depression, initially presented in 2020 with mediastinal and hilar lymphadenopathy. FDG-PET imaging demonstrated extensive hypermetabolic mediastinal and abdominal lymphadenopathy with pulmonary lesions, raising suspicion for active sarcoidosis versus lymphoproliferative disease. Subsequent mediastinoscopy confirmed non-caseating granulomas, establishing the diagnosis of pulmonary sarcoidosis. She received a three-month course of corticosteroids, after which treatment was discontinued, and she remained asymptomatic but was subsequently lost to follow-up.

**Figure 1:**
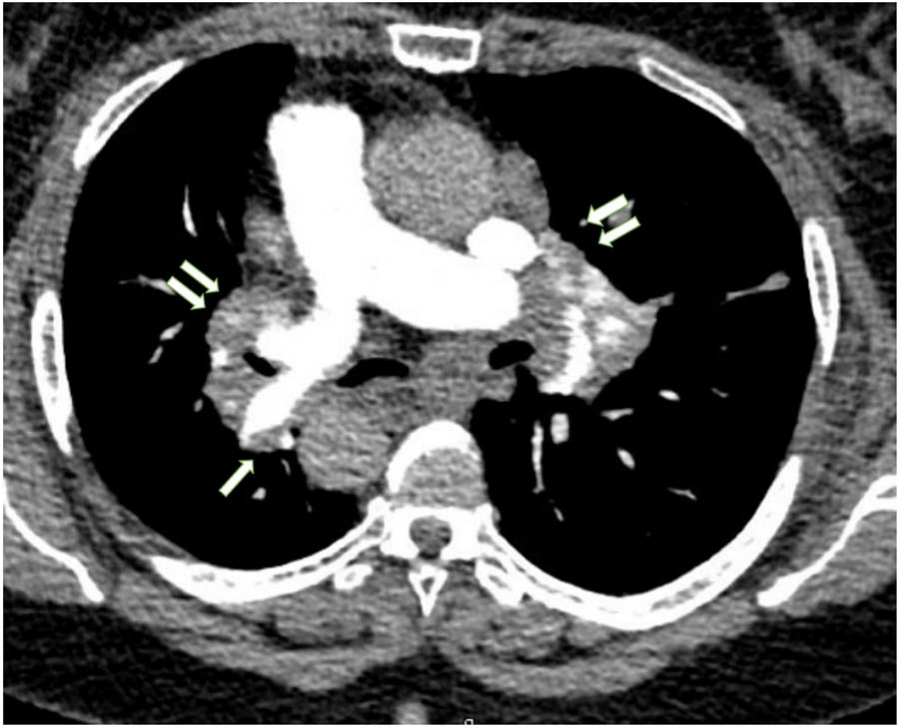
CT pulmonary angiography. Contrast-enhanced CT pulmonary angiography demonstrating bilateral hilar and mediastinal lymphadenopathy (paired arrows) and a right lower lobe pulmonary arterial filling defect (single arrow) consistent with acute pulmonary embolism.

She re-presented in 2026 with acute dyspnea, right leg pain, palpitations, and dizziness. She was hemodynamically stable, with normal cardiac biomarkers and no echocardiographic evidence of right ventricular strain. CT pulmonary angiography (Fig. 1) acute segmental and subsegmental pulmonary emboli in the right lower lobe pulmonary arteries with extensive mediastinal and hilar lymphadenopathy and multiple bilateral pulmonary nodules.

**Figure 2:**
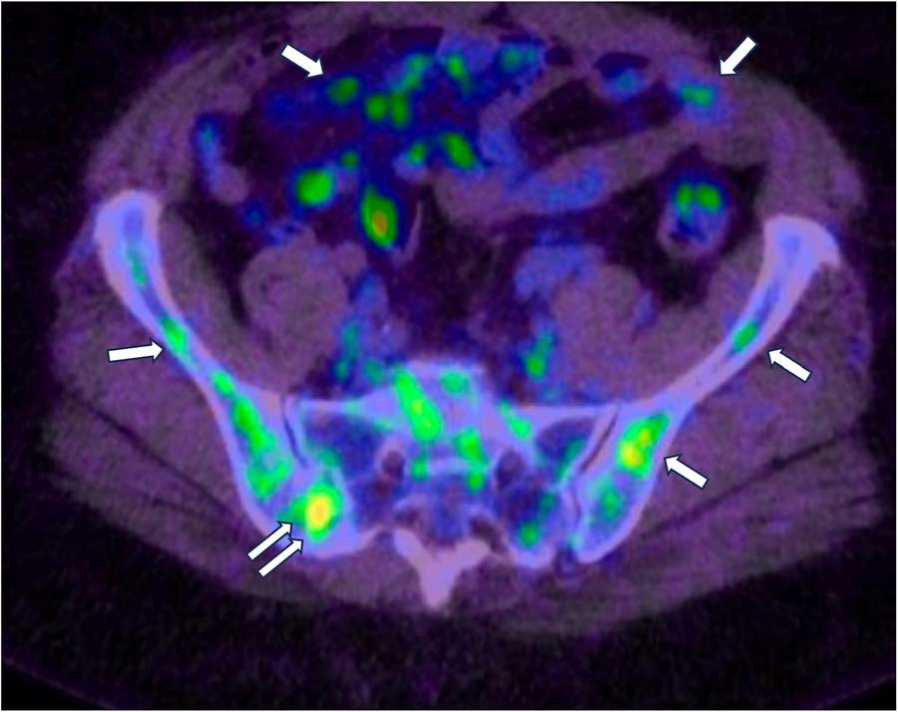
Axial FDG-PET/CT. Axial FDG-PET/CT demonstrating multiple intensely hypermetabolic osseous lesions involving the pelvis and right iliac bone (two arrows, SUVmax 12.3), together with widespread FDG-avid nodal and additional osseous lesions (single arrows), highly suspicious for disseminated metastatic disease.

FDG-PET/CT demonstrated widespread intensely hypermetabolic disease involving thoracic, abdominal and pelvic lymph nodes, pulmonary nodules, liver, spleen and multifocal skeletal lesions. Representative findings included a left lower lobe pulmonary nodule measuring 1.5 cm (SUVmax 11.7), a prevascular mediastinal lymph node (SUVmax 16.6), a left periaortic lymph node (SUVmax 12.6), and an intensely FDG-avid right iliac osseous lesion (SUVmax 12.3) (Fig. 2). Additional FDG-avid skeletal foci were identified within the spine, pelvis and proximal long bones. The extensive distribution and intense FDG avidity raised strong suspicion for disseminated metastatic malignancy or lymphoma.

**Figure 3:**
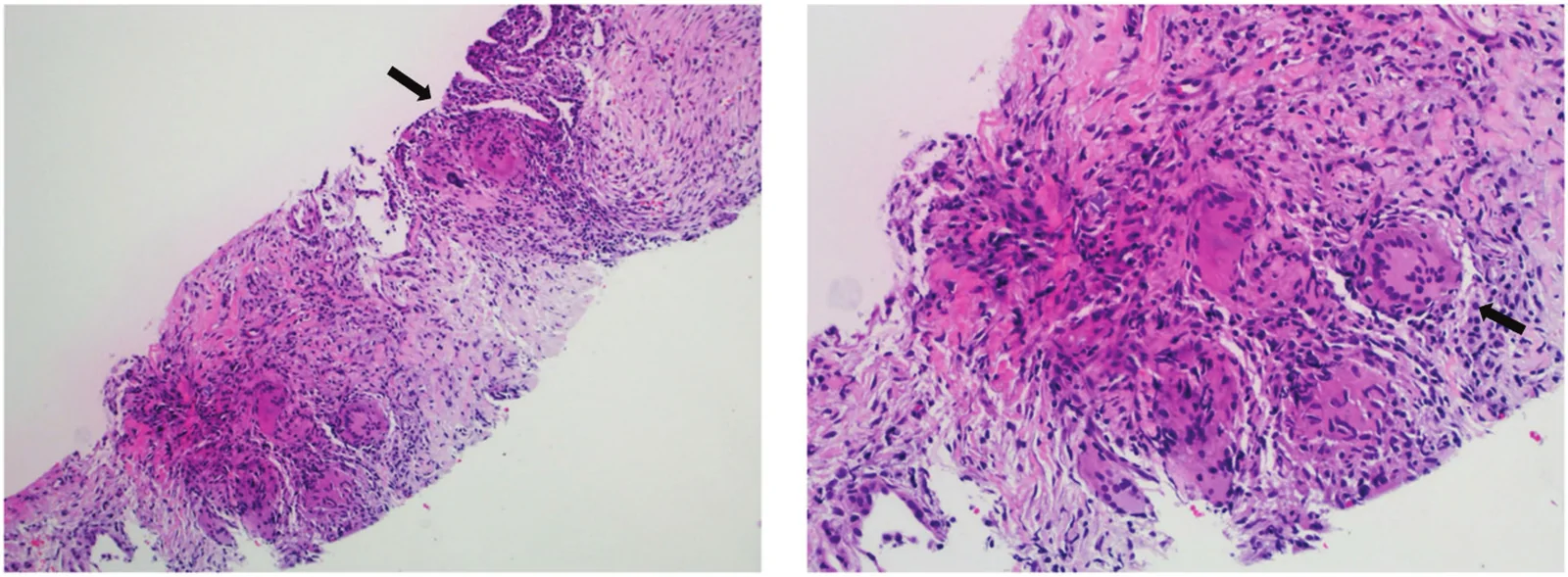
Right iliac bone biopsy. Histopathological examination of the right iliac bone biopsy (hematoxylin and eosin stain; left, ×10; right, ×40) demonstrating non-necrotizing granulomatous inflammation (black arrows) without evidence of malignancy or infection, consistent with osseous sarcoidosis.

CT-guided biopsy of the iliac lesion (Fig. 3) showed non-necrotizing granulomatous inflammation with negative microbiological stains for fungal and mycobacterial organisms, and no evidence of malignancy, confirming osseous sarcoidosis. She was commenced on therapeutic anticoagulation with apixaban for pulmonary embolism. Given mild symptoms, stable pulmonary function testing, absence of organ-threatening disease, and multidisciplinary consensus evaluation, immunosuppressive therapy was not initiated, and the patient was managed conservatively with close follow-up. Cardiac evaluation excluded cardiac sarcoidosis. The patient remains clinically stable under multidisciplinary follow-up.

## Discussion

This case illustrates the diagnostic challenge posed by multisystem FDG-avid sarcoidosis closely mimicking disseminated metastatic malignancy. Extensive hypermetabolic involvement of lymph nodes, lungs, liver, spleen and multiple skeletal sites with high SUV values initially raised strong suspicion for advanced neoplastic disease. However, active granulomatous inflammation in sarcoidosis may demonstrate marked FDG uptake overlapping substantially with malignant processes on PET imaging [2].

Osseous sarcoidosis is an uncommon manifestation, occurring in approximately 3%–13% of patients with sarcoidosis, although prevalence varies depending on the population studied and imaging modality used. It is frequently asymptomatic and may therefore be underrecognized. Radiologically, multifocal osseous lesions can closely mimic skeletal metastases, making histopathological confirmation essential for an accurate diagnosis [3, 4]. In this case, multifocal FDG-avid skeletal lesions involving the spine, pelvis and proximal long bones together with visceral involvement strongly favored disseminated malignancy on imaging. Histological evaluation of the FDG-avid iliac lesion was therefore essential to exclude malignancy and confirm multisystem sarcoidosis.

Although osseous involvement reflects multisystem disease, it does not necessarily confer a worse overall prognosis. Clinical outcomes are primarily determined by the extent and severity of involvement of vital organs, particularly the lungs, heart, and nervous system, rather than skeletal lesions alone. With appropriate management, many patients with osseous sarcoidosis achieve favorable long-term outcomes, although relapses and chronic disease may occur [5].

Treatment of osseous sarcoidosis is individualized according to symptom burden and the extent of organ involvement. Corticosteroids remain the first-line therapy for symptomatic or organ-threatening disease, while steroid-sparing immunosuppressive agents, such as methotrexate, may be considered in patients requiring prolonged treatment or with refractory disease. In our patient, conservative management with close multidisciplinary follow-up was considered appropriate because she had mild symptoms, preserved organ function, and no cardiac or neurological involvement [6].

An additional notable feature was the concurrent pulmonary embolism. Sarcoidosis has been associated with an increased risk of venous thromboembolism, likely related to chronic systemic inflammation and endothelial dysfunction [7].

## Conclusion

Multisystem sarcoidosis may closely mimic disseminated metastatic malignancy on FDG-PET/CT, particularly in the presence of multifocal skeletal and visceral involvement. Histological confirmation remains essential to establish the diagnosis and avoid inappropriate oncological management.

## Data Availability

All data and materials related to this case report are available from the corresponding author upon reasonable request.

## CARE guidelines

This case report was prepared in accordance with the CARE guidelines.
